# Competitive Market Analysis of Transplant Centers and Discrepancy of Wait-Listing of Recipients for Kidney Transplantation

**Published:** 2015-11-01

**Authors:** P. S. Cho, R. F. Saidi, C. J. Cutie, D. S. C. Ko

**Affiliations:** 1 Department of Urology, Massachusetts General Hospital, Harvard Medical School, Boston, MA, USA; 2 Division of Organ Transplantation, Department of Surgery, Alpert Medical School of Brown University, Providence, RI, USA; 3 *Department of Surgery, Division of Transplantation, Massachusetts General Hospital, Harvard Medical School, Boston, MA, USA*

**Keywords:** Kidney transplantation, Competition, Allocation, Wait-list, Market analysis

## Abstract

**Background:**

There are over 250 kidney transplant programs in the USA.

**Objective:**

To determine if highly competitive regions, defined as regions with a higher number of transplant centers, will approve and wait-list more end-stage renal disease (ESRD) candidates for transplant despite consistent incidence and prevalence of ESRD nationwide.

**Methods:**

ESRD Network and OPTN data completed in 2011 were obtained from all transplant centers including listing data, market saturation, market share, organs transplanted, and ESRD prevalence. Herfindahl-Hirschman Index (HHI) was used to measure the size of firms in relation to the industry to determine the amount of competition.

**Results:**

States were separated into 3 groups (HHI<1000 considered competitive; HHI 1000–1800 considered moderate competition; and HHI>1800 considered highly concentrated). The percentage of ESRD patients listed in competitive, moderate, and highly concentrated regions were 19.73%, 17.02%, and 13.75%, respectively. The ESRD listing difference between competitive versus highly concentrated was significant (p<0.05).

**Conclusion:**

When there is strong competition without a dominant center as defined by the HHI, the entire state tends to list more patients for transplant to drive up their own center’s market share. Our analysis of the available national data suggests a discrepancy in access for ESRD patient to transplantation due to transplant center competition.

## INTRODUCTION

The shortage of donor kidneys and the growing waiting lists have long been unsolved problems in transplantation [1, 2]. The allocation of these organs has consequently been a clinical and ethical challenge that has yet to see a perfect solution. In the US, there are over 250 kidney transplant programs grouped geographically by adjacent states into 11 organ donation regions to promote sharing of deceased donor kidneys [3]. The Organ Procurement Transplant Network (OPTN) has enacted several iterations of the Final Rule since 2000 in an attempt to provide better and more efficient allocation [4-6].

We hypothesize that highly competitive regions tend to approve and wait-list more end-stage renal disease (ESRD) candidates for transplant despite consistent incidence and prevalence of ESRD nationwide. To assess the competitiveness of transplant markets, the Herfindahl-Hirschman Index (HHI) has been utilized to assess for market competitiveness, including within the health care industry [7-9]. We applied this index as part of our efforts to evaluate the available public data from all transplant centers, including listing data, market saturation, market share, organs transplanted, and ESRD prevalence to evaluate if highly competitive states will have different rates of recipient listing for deceased donor renal transplants in the US.

## MATERIALS AND METHODS

Data Sources

ESRD prevalence was obtained through available published data from the ESRD Network Programs through Centers for Medicare and Medicaid Services (CMS). Data through 2010 were available and utilized. The available public data from all transplant centers in the USA including listing data and numbers of organs transplanted were obtained through the OPTN and the United Network of Organ Sharing (UNOS) [3]. For purposes of comparison, data from 2011 were utilized for calculation. Information from these sources was utilized to determine percentage of patients with ESRD listed for renal transplantation and to determine market share for deceased donor kidney transplants.

Herfindahl-Hirschman Index

The HHI is a commonly accepted standard of economic measure of market competition and concentration [7-9]. The index is calculated by the squaring the market share of each center competing in a market or region, and then summing the resulting numbers:

The range for HHI is 0 to 10,000. An index of <100 denotes a highly competitive market, while an index of <1500 suggests a non-concentrated, competitive market. An HHI between 1500 and 2500 indicates moderate concentration and an index of >2500 suggests high concentration. An index of 10,000 denotes a monopoly or single dominant center.

## RESULTS

Competition of Centers Within UNOS Regions

The HHI was applied and calculated for the 11 UNOS regions (Table 1). The resulting HHI for these regions ranged from 594 to 1319. As indices in all regions were <1500, all UNOS regions were contained competitive markets. No region had indication of a dominant center or concentration.

**Table 1:** Competition by HHI within UNOS regions

Region	State	Numbers of centers	HHI
1	ME, NH, MA, CT, RI	14	854
2	NJ, PA, DE, MD, DC, WV	34	594
3	AL, AR, FL, GA, LA, MS, PR	26	732
4	OK, TX	31	722
5	AZ, CA, NV, NM, UT	37	642
6	AK, HI, ID, MT, OR, WA	10	1319
7	IL, MN, ND, SD, WI	22	941
8	CO, IA, KS, MO, NE, WY	21	1019
9	NY, VT	16	1089
10	IN, OH, MI	24	886
11	KY, NC, SC, TN, VA	24	651

Competition of Centers Within States

**Figure 1:**
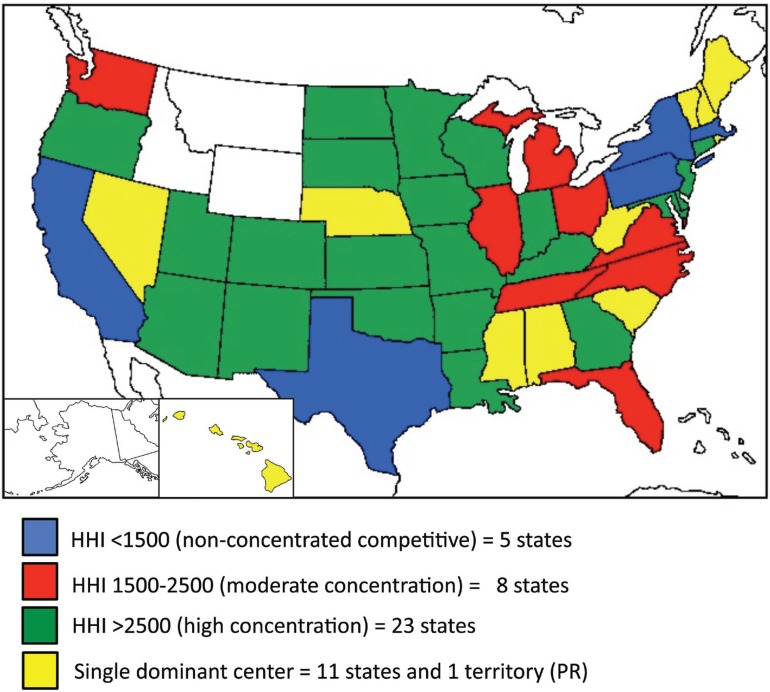
Competition by HHI within states

The HHI was applied to centers within a given state. All 50 states and one territory (Puerto Rico) could be grouped into non-concentrated competitive (HHI<1500), moderately concentrated (1500≤HHI≤2500), highly concentrated (HHI>2500), or single-center (HHI 10,000) markets for transplant centers (Fig 1). Eleven states and one territory (Puerto Rico) were single-center markets. Twenty-three states were categorized as highly concentrated markets. Eight states were found to be moderately competitive markets, while five states were non-concentrated competitive systems. These groups were utilized for purposes of subsequent comparison.

Comparison of Listing of Patients

States were grouped based on HHI, and percentage of ESRD patients listed for kidney transplant and of patients receiving deceased donor kidney transplants were determined for each state (Tables 2–5). When comparing states based on these four groups, with increasing competition and decreasing concentration of centers, an increasing number of transplant centers was observed. States with HHI <1500, denoting high competition and low concentration, had an average of 18.2 transplant centers per state. In comparison, moderately concentrated states and highly concentrated states had an average of 8.7 and 3.9 centers per state, respectively. States with high competition and HHI <1500 had an average percentage of ESRD patients listed for transplant of 22.5%. In comparison, states that were moderately competitive or highly competitive listed 15.2% and 15.9% of patients, respectively. Single-center states listed an average of 9.7% of patients. These differences were statistically significant (Table 6). In terms of percentage of ESRD patients transplanted with deceased donor kidneys, in competitive, moderately concentrated, and highly concentrated states, the average percentage of patients transplanted was similar at 2.1%. In single-center states, the percentage appeared to be slightly lower at 1.4%, but this was not statistically significant.

**Table 2:** ESRD patients listed and transplanted, HHI <1500

State	HHI	Number of centers	Average market share/center	%ESRD listed	%ESRD transplanted
MA	1250	9	11.1	20.2	2.4
PA	996	18	5.56	26.6	2.7
TX	857	25	4.2	20.1	1.7
CA	957	23	4.3	24.9	1.7
NY	1154	16	6.7	20.9	1.8
Average	1042.8	18.2	6.4	22.5	2.1

**Table 3:** ESRD patients listed and transplanted HHI 1500–2500

State	HHI	Number of centers	Average market share/center	%ESRD listed	%ESRD transplanted
IL	2250	9	11.1	16.6	1.3
TN	2148	9	11.8	18.1	2.5
OH	1778	11	9.1	12.3	1.6
VA	2155	6	16.7	17.9	1.9
WA	2342	5	20	16.9	2.5
MI	2298	10	10	12.2	1.6
NC	2253	5	20	17.1	3.2
FL	1834	11	9.1	10.4	2.3
Average	2132.3	8.3	13.5	15.2	2.1

**Table 4:** ESRD patients listed and transplanted, HHI >2500

State	HHI	Number of centers	Average market share/center	%ESRD listed	%ESRD transplanted
CT	5805	2	50	18.9	1.8
NJ	3022	6	20	17.3	1.4
DE	7066	2	50	21.9	0.9
DC	4321	5	20	43.3	5.2
MD	5000	2	50	13.7	2.1
AR	4248	3	33.3	3.2	1.8
GA	3310	5	20	15.4	1.4
OK	3458	6	16.7	9.9	1.9
AZ	3652	7	14.3	15.1	1.7
NM	5053	2	50	10.7	1.6
UT	4822	4	25	13.5	2.7
OR	3946	3	33.3	14.7	2.2
ND	5823	2	50	12.3	1.7
SD	3746	2	50	19.2	1.5
WI	4614	4	25	17	2.4
CO	3801	4	25	27.1	2.9
IA	3679	4	25	11.9	2.4
KS	6553	2	50	12.5	2.4
MO	3520	10	10	11.5	2.5
IN	6290	3	33.3	10.9	1.5
LA	4234	4	25	13.8	2
KY	4943	3	33.3	7.9	2.1
MN	3123	5	20	24.3	2.1
Average	4524.2	3.9	31.7	15.9	2.1

**Table 5:** ESRD patients listed and transplanted HHI 10,000 (Single-Center)

State	%ESRD listed	% ESRD transplanted
ME	6.1	1.2
NH	7	1.7
RI	14.1	1.5
WV	4.5	1
AL	32.9	1.5
MS	1.4	0.5
PR	6.8	1.2
NV	2.3	1.2
HI	11.3	1.1
VT	11.9	3.5
NE	9.5	4.6
SC	8.6	1.4
Average	9.7	1.7

**Table 6:** Comparison of Listing and Transplantation

Parameter	Competitive	Moderately concentrated	Highly concentrated	Single center
HHI	<1500	1500–2500	>2500	10,000
Number of state	5	8	23	12
Average number of transplant centers	18.2	8.7	3.9	1
Average % ESRD patients listed	22.5	15.2	15.9	9.7
Average % ESRD patients transplanted with DD kidneys	2.1	2.1	2.1	1.4

## DISCUSSION

In the USA, transplantation has always been considered a top-tier medical and surgical service line for a hospital. The ability to treat end-organ failure with organ replacement therapy, in some investigative cases successfully even without immunosuppression, remains on the cutting edge of medicine [10, 11]. Transplantation brings the most medically complex patients for care to a hospital, generating significant immediate and downstream revenue. Because of the need for sustainability in any medical system in a free market, service lines that are revenue generators are considered to be highly desirable. Consequently, transplantation programs continue to try to expand despite being in a supply limited market space. It is clear that there exists significant competition among the transplant centers for the market share.

As the prevalence of ESRD increases, there has been a parallel increase in the size of the deceased-donor kidney transplantation wait-list [12]. Kidney transplantation is the treatment of choice for these patients for survival, quality of life and costs [13]. The number of patients listing for kidney transplantation is increasing but at a seemingly disproportionate rate than the prevalence of ESRD patients on renal replacement therapy. One strategy utilized to address the critical shortage of kidneys for transplantation is the expansion of the deceased donor kidney pool to include those kidneys which might have been deemed unsuitable for use in the past [14-17]. Despite all these strategies, the number of organs available and kidney transplantations has been stagnant, if not declining in the past few years [2, 18].

Patients with ESRD from one area of the USA can be evaluated and may be listed in multiple regions in the country. Such activity has raised concerns about equity to access for transplantation. Moreover, in recent years, high-profile patients with end-stage organ disease research where they may get an organ “faster” and subsequently travel to those centers to obtain an organ transplant [19]. Incidences of this practice has further fueled the call for reevaluation of the fairness and transparency of organ allocations practices nationwide.

These revelations are not new, as this “gaming” of the system has been noted previously prior to the enactment of regulations, specifically in regards to the allocation of heart and liver allografts [20, 21]. Prior to the enactment of the Final Rule in 2000, gaming and its relation cardiac transplant listing were evaluated. In this particular report, increased competition between centers resulted in increased number of patients being listed in the most severe category. Following the enactment of new regulations, this effect was mitigated. Nonetheless, this emphasizes the need for ongoing monitoring and perhaps utilizing and enforcing sanctions in the future to discourage this gaming the system for transplantation.

Similarly, concerns are being raised in regards to renal transplantation competition as the number or wait-list recipients and the number of transplant programs have increased while donor supply remains stable [18, 22]. As each program mandates growth in clinical volume to remain profitable or solvent in today’s highly contentious medical economy, each center must strategize to assess their opportunities for growth. However, confined by the current allocation scheme and a stable organ supply model, the distribution of organs favors those centers with larger wait-lists. The larger waiting lists statistically will draw more organs for the individual transplant center when UNOS generates a recipient list for an available organ. The increasing number of patients on waiting lists further speaks to the aggressiveness of a center and potentially transplantation of less than ideal candidates [23].

Competition analysis in economics in medical care is also not a new phenomenon. In 1933, statistician Horace Secrist construed the theory of organizational mediocrity where it parallels the concept of regression to the mean. Whenever competition increases in a market segment, volume changes can be favorable for some while adversely affecting others in the short-term. However, there is a very predictable regression back to the *status quo* prior to the perturbation that can be observed. This has been observed in transplantation competition when in Region I of UNOS as a change in transplant leadership consistently led to a center increasing their clinical kidney transplant volume in a short time. However, the clinical volume returned to a stable equilibrium within 24 months [24]. Although this analysis was not designed to answer whether competition itself leads to overall increases in volume or demand in a cohort of competing programs, it begs the question of whether competition amongst centers impacts listing of patients to durably increase the overall transplant volume.

The goal of our study was to determine whether listing for kidney transplantation in the setting of competition results in an overall increase, specifically whether these economic pressures indirectly medical decision-making. In an era of increasing regulatory and governing bodies in transplantation in the US, the CMS has mandated protocols and policies for determining the eligibility criteria for patient listing with UNOS [25]. Although there are general medical guidelines for transplant eligibility as determined by evidence-based medicine, the CMS mandate does not call for national uniformity, but only center specific protocols. Against common wisdom, this variability allows the patients with ESRD to be listed at one transplant center while their same medical comorbidities may exclude them from another. 

The HHI is a measure of competition among players in a particular industry or market. It is a commonly accepted measure of market concentration and the US Department of Justice uses the HHI for evaluating mergers and antitrust concerns [26]. The application of the HHI to transplant centers within states provides a novel perspective to health care competition. Our results indicate that when a competitive market exists in which there are many centers in competition and without a dominant center, a higher percentage of patients tend to be listed for transplant. However, despite this difference, the percentage of ESRD patients receiving deceased donor transplants as a result of these lists was not different despite the competitiveness of the market for these organs. Centers in such areas may increase the size of waiting lists in order to drive clinical volume and to maintain competitiveness in securing a limited resource.

This difference observed suggests a discrepancy in access of ESRD patients and their ability to be listed for transplantation as a result of center competition. Therefore, this raises concern of inequitable access to the possibility of renal transplantation for ESRD patients nationwide, as patients in less competitive markets seem less likely to be considered for transplantation. Our study, using available public data and standard methodology for assessing market competition, provides a preliminary suggestion that there may be a geographic inequity to access to renal transplantation. With different rates of ESRD patients being wait-listed for transplant and relatively similar incidences of ESRD across states, it seems that more candidates are being considered in competitive markets. This raises concern that this may not be an effective allocation of a limited resource, as it may exclude patients who may derive greater long-term benefit from transplantation. Such economic factors may ultimately affect costs of health care delivery and thus merit consideration when balanced with short-term costs of center competition.

The intent of our study was to identify whether a standard methodology to access competition in market competition can be applied to renal transplantation to show a difference rate of listing of ESRD patients for transplantation. Our study combined competition indices based on states, and recognizing that there are significant differences by donor service area (DSA) within or across each state. Understandably, patient flows to transplant centers do not stop at state lines as they can be listed at multiple centers across the USA. While the use of states is arbitrary, it is the only publically available data on ESRD prevalence that is accessible through the ESRD networks programs and that data are confined by states with access of prevalence by state population data. We also recognized that a potential pitfall of our initial observation was that the data were not adjusted for demographic differences in the ESRD population and presenting unadjusted data might be misleading. However, we were not evaluating this based on demographics but rather the percentage of patients with ESRD observed a defined population area that was listed. We observed a difference in listing rate as it correlated to how strong market competition was for that area. 

It may also be useful to look at differences in patient characteristics as a function of competition. Are centers in competitive regions listing older, more marginal candidates? Do the single center (low competition) areas list only younger patients? However, that would call for a large multi-center collaborative study where specific recipient medical conditions can be retrospectively analyzed in detail. Furthermore, an evaluation of organ export from low competitive regions and whether center competition increases aggressiveness for organ acceptance into high competitive areas may be necessary to understand the nature supply and demand that is beyond the scope of our study. These are unexplored areas of transplant market competition that deserve future detailed collaborative analysis as one considers changes to allocation of a scare and rationed resource.

Scarcity will lead to competition. Competition can lead to many socially desirable outcomes such as more choices for the patients, lower prices and higher productivity. The transplant centers have to behave under pressures and influences of fiscal realities and human resource utilization. Since transplant centers must perform transplantation procedures at rates high enough to meet their fixed costs, and seek incremental profits with each additional transplant, each center must maximize measures that preserve their market share. The centers in competitive regions tried to list more patients to increase their chance to capture the limited organ supply.

Despite best intentions in establishing national guidelines for equity and justice for access to organ transplantation, effects of competition and medical economics are factors that may impact delivery of medical care. Although multiple centers in a geographic region may theoretically provide better access for patients, there may be unintended consequences affecting waitlists and ultimately impacting a patient’s ability to receive a transplant. 

## Note

The abstract of this manuscript was presented at the American Urological Association 2013 Annual Meeting in San Diego, CA, Competitive Market Analysis of Transplant Centers and Discrepancy of Patient Access to Kidney Transplantation. *J Urol *2013;**189 (4, Suppl)**:e946-7.

## References

[1] 2009 Annual Report of the U.S. Organ Procurement and Transplantation Network and the Scientific Registry of Transplant Recipients: Transplant Data 1999-2008. U.S. Department of Health and Human Services, Health Resources and Services Administration, Healthcare Systems Bureau, Division of Transplantation, Rockville, MD.

[2] Klein AS, Messersmith EE, Ratner LE (2010). Organ donation and utilization in the United States, 1999-2008. Am J Transplant.

[3] UNOS, United network for organ sharing.

[4] Organ procurement and transplantation network (2000). The Final Rule-Federal Regulation for the operation of the OPTN. DoHaH. Services. hhttp://www.optn.transplant.hrsa.gov/policiesAndBylaws/final_rule.asp.

[5] Brown RS, Higgins R, Prutt TL (2009). The Evolution and Direction of OPTN Oversight of Live Organ Donation and Transplantation in the United States. Am J Transplant.

[6] McDiarmid SV, Pruett TL, Graham WK (2008). The Oversight of Solid Organ Transplantation in the United States. Am J Transplant.

[7] Hyman DA, Kovacic WE (2004). Monopoly, monopsony, and market definition: an antitrust perspective on market concentration among health insurers. Health Aff.

[8] Nauenberg E, Alkhamisi M, Andrijuk Y (2004). Simulation of a Herfindahl-Hirschmanindex without complete market share information. Health Econ.

[9] Reuter J, Gaskin D (1997). Academic Health Centers in Competitive Markets. Health Affairs.

[10] Kawai T, Cosimi AB, Spitzer TR (2008). HLA-Mismatched enal Transplantation Without Maintenance Immunosuppression. N Engl J Med.

[11] Kawai T, Sachs DH, Sykes M, Cosimi AB (2013). Immune Tolerance Network. HLA-Mismatched Renal Transplantation Without Maintenance Immunosuppression. N Engl J Med.

[12] Van Walraven C, Manuel DG, Knoll G (2014). Survival Trends in ESRD Patients Compared With the General Population in the United States. Am J Kidney Dis.

[13] Wolfe RA, Ashby VB, Milford EL (1999). Comparison of mortality in all patients on dialysis, patients on dialysis awaiting transplantation, and recipients of a first cadaveric transplant. N Engl J Med.

[14] Chapman J, Bock A, Dussol B (2006). Follow-up after renal transplantation with organs from donors after cardiac death. Transpl Int.

[15] Saidi RF, Elias N, Kawai T (2007). Outcome of kidney transplantation using expanded criteria donors and donation after cardiac death kidneys: realities and costs. Am J Transplant.

[16] Whiting JF, Delmonico F, Morrissey P (2006). Clinical results of an organ procurement organization effort to increase utilization of donors after cardiac death. Transplantation.

[17] Merion RM, Ashby VB, Wolfe RA (2005). Deceased-donor characteristics and the survival beneﬁt of kidney transplantation. JAMA.

[18] Saidi RF, Markmann JF, Jabbour N (2012). The Faltering Solid Organ Donor Pool in The United States (2001-2010). World J Surg.

[19] Grady D, Meier B (2009). A Transplant That Is Raising Many Questions. The New York Times.

[20] Scanlon DP, Hollenbeak CS, Lee W (2004). Does competition for transplantable hearts encourage ‘gaming’ of the waiting list?. Health Aff.

[21] Halidorson JB, Paarsch HJ, Dodge JL (2013). Center Competition and Outcomes Following Liver Transplantation. Liver Transplantation.

[22] Axelrod DA, McCullough KP, Brewer ED (2010). Kidney and pancreas transplantation in the United States, 1999-2008: the changing face of living donation. Am J Transplant.

[23] Garonzik-Wang JM, James NT, Weatherspoon KC (2012). The aggressive phenotype: center-level patterns in the utilization of suboptimal kidneys. Am J Transplant.

[24] Saidi RF, Khaksari S, Ko DSC (2014). Effect of staff migration on kidney transplant volume in region 1. Progress in Transplantation.

[25] Centers for medicare and Medicaid services Transplant Program Application Requirements.

[26] Horizontal Merger Guidelines.

